# Inhumation

**Published:** 1880-04

**Authors:** 


### INHUMATION.

People dislike to accept any innovation upon their usual and accepted customs. Inhumation of human bodies has been so long practiced, and the usual ceremonies attending a funeral have been so satisfactory to those who refuse to give the matter any thought, or if they do, who care little or nothing about its influence upon the health of the living, that it becomes difficult to arouse a public sentiment against the practice. It has been shown, and medical men are universally agreed upon the subject, that to inhumation can be traced many of the diseases which increase the opportunities for this barbarous custom. Time and again, have epidemics of diphtheria, typhoid fever, and other contagious and fatal diseases been traced to the drinking of foul water that has emanated from the festering bodies in our grave-yards, and from the decomposing animal and vegetable matter whose drippings find their way into our springs and wells. It has been demonstrated, and the fact has been widely published, that bodies having died from these infectious diseases not only poison the air and water with germs the result of simple putrefaction, as they lie festering in the ground, but the more dangerous ones also that arise from the specific disease of which the body died.

Earth is regarded as a deodorizer. So it is, when dry and pulverized. But, deodorization does not include disinfection. Because the rotting body is not recognizable by our olfactories, is no evidence that it is not reeking in nastiness and poison. Look at a grave a week or two after burial of a body. See how the mound has sunken below the level of the surrounding earth forming a basin in which the rain pours to saturate the body at the bottom of the pit. Rearrange your mound; still the drenching rains will find an easy passage through the unpacked soil, to trickle upon the decaying body and from thence percolate, with its death-dealing germs, to the water courses and wells in the valley below. All this is known to the majority of reading and thinking people. They are familiar, too, with the fact that a beautiful, inexpensive and entirely wholesome remedy for all this exists in cremation. But they shrink from the process because they dread to think of burning the bodies of their loved ones. Burning! Why, did you ever stop to think of the horrible alternative ? Did you ever see a grave opened, to witness the loathesome, festering nastiness that lies at the bottom in which wiggling worms and horrid bugs feast and find a hiding place beneath! Compare this slow, filthy combustion of the body to the beautiful, pure, ever cleanly and wholesome method of cremation. Decay, though unobserved, is but a slow and nasty process of burning. It is a weak and imbecile sentimentality that will defend it. Why take years to do that which can be cleanlier accomplished in two hours ? Why subject the loved form to a pit of reeking filth and rottenness when cremation offers a pure and undefiled disposal of it?

Cremation presents a speedy, cleanly, inexpensive, wholesome and beautiful method of disposing of our dead. It need not be in the slightest degree shocking, even if observed, to the most sensitive organization. Let a little chapel and crematory be erected in our cemetery, where those who prefer cremation to inhumation can avail themselves of this sensible disposition of their dead, where the body can be wrapped in a white sheet placed lovingly, and with a prayerful benediction, upon the iron cradle to be conveyed by trusted friends to the purifying influence of the heated retort. Then, while the body is being reduced to ashes, the friends are congregated in the adjoining chapel listening to pleasant words about the deceased from the clergymen, and to prayers and songs appropriate to the occasion. When the ashes are brought you in an ornamental urn provided for the purpose, they can then be buried with the satisfaction of knowing that they can never be defiled with rottenness to poison the living, nor be disinterred to satisfy the quibblings of an insurance company, nor become an object of traffic for body snatchers. Can there be any doubt in the mind of any sensible person as to which is the most desirable method ?

We publish elsewhere a letter from the executor of Dr. LeMoyne, giving information touching the cost of building a furnace and of cremating a body.* We have planned a crematory and chapel that will answer the purpose of our city, and need not cost more than one thousand dollars. The cost of consuming a body—including pay of attendant and fuel, should not exceed twenty dollars. We believe that if an undertaker like Mr. Robinson would erect such an establishment in our city, a plenty of people could be found who would be willing, nay glad, to dispose of their dead by cremation, and suitably remunerate him for the outlay. The process need only be seen to commend it to every intelligent person.

*This letter, with an excellent one from Dr. Gleason, both of which are in type, have been crowded out.

